# Supplementary material for: Getting More Out of Biomedical Documents with GATE's Full Lifecycle Open Source Text Analytics
Source: PLoS Comput Biol. 2013 Feb 7;9(2):e1002854. doi: 10.1371/journal.pcbi.1002854 (PMC3567135; doi:10.1371/journal.pcbi.1002854)
Supplement: Dataset S1 — GATE software. Dataset S1 bundled with this paper contains a distribution of GATE (or see http://gate.ac.uk/download/). (TGZ) [file pcbi.1002854.s001.tgz › plos-gate/plugins/Lang_Chinese/resources/grammar/note4gram.doc]

Date:Lookup.majorType==date (This includes items in the following lists:Century/date/date_spec/day/decade/festival/month/season/year/year_spec)adbc + centuryadbc + century + century_suffixdecade +decade_suffixcentury +decadecentury +decade+decade_suffixmonth + month_suffixmonth + dateyear + month + dateyear + month + date +dayyear + seasonyear + festivalyear + year_suffixyear_spec+year_suffixyear_spec+monthdigits + year_key (digits consists of 1 to 4 items from the digit.lst)digits + year_key + year_suffix (digits consists of 1 to 4 items from the digit.lst)adbc + yearadbc + digits + year_key Location:Lookup.majorType==location (This includes items in the following lists:city_abbrev/city_china/city_world/country/country_abbrev/county/county_abbrev/facility/loc_relig/ mountain/province/province_abbrev/region/river/state/town)Token.String includes an item in loc_key.lstLocations can be combined together to form a location. Money:number + currency_unitmoney_prefix + numbermoney_prefix + number + currency_unitnumber_unit + currency_unitOrganization:Lookup.majorType==organization (This includes items in the following lists:association/ company/ company_CHN/ company_media/ government/ org_sport/ organization/ university)Token.String includes an item in org_key.lstorg_prefix + organizationPeople:Lookup.majorType==person (This includes items in the following lists:person/person_relig/surname_foreign)Chinese namesurname + firstname (1 or 2 characters) (title appears before surname or after firstname)surname + person_endingsurname + firstname (1 or 2 characters) ( person_ending appears after firstname)Foreign nameforename + “.” + s_name (forename consists of items from male.lst or female.lst)forename + s_name (forename consists of items from male.lst or female.lst)s_name (title appears before or after)s_name (person_ending appears after)Percent:Lookup.majorType==percent (This includes items in percent.lst)percent_pre + numbernumber + percent_endNumber:Lookup.majorType==number (This includes items in number.lst)zero + point + numberdigit + number_unitdigit + point + numberdigit + point + zero + numberdigit + point + digit + numbernumber + point + zero + numbernumber + zero + numbernumber + more + number_unitnumbers can be combined together to form a number.The longest string gets the highest priority.For example,century +decadecentury +decade+decade_suffixboth construct a ‘DATE’, but ‘century +decade+decade_suffix’ gets the higher priority.Another example, if locations are not separated words or symbols, they (the whole string) must be marked as a location.
